# Categorization of wheat genotypes for phosphorus efficiency

**DOI:** 10.1371/journal.pone.0205471

**Published:** 2018-10-17

**Authors:** Hafiz Muhammad Bilal, Tariq Aziz, Muhammad Aamer Maqsood, Muhammad Farooq, Guijun Yan

**Affiliations:** 1 Institute of Soil and Environmental Sciences, University of Agriculture, Faisalabad, Pakistan; 2 UWA School of Agriculture and Environment, The University of Western Australia, Perth, Australia; 3 Department of Agronomy, University of Agriculture, Faisalabad, Pakistan; 4 Department of Crop Sciences, College of Agricultural and Marine Sciences, Sultan Qaboos University, Al-Khoud, Oman; Institute of Genetics and Developmental Biology Chinese Academy of Sciences, CHINA

## Abstract

Production of phosphorus efficient crop cultivars can increase food productivity and decrease environmental pollution. Categorization of existing germplasm is a prerequisite to develop P efficient crop cultivars. For first experiment, 30 wheat genotypes were grown in hydroponics with two P levels (i.e., deficit, 20 μm KH_2_PO_4_ and adequate, 200 μm KH_2_PO_4_). Genotypes differed significantly for various P efficiency parameters. Two genotypes (Dirk and Bhakkar-02) showed < 25% decrease in growth at P deficiency. Genotype Seher-06 proved to be inefficient. Twelve selected genotypes based on the first experiment were sown in soil with two P levels (0 and 30 mg P kg^-1^) till maturity. As expected, genotypes differed for grain yield at both P levels. The efficient cultivars selected on the basis of both absolute and relative dry matter production at both P levels such as Dirk. Genotypes were grouped into three, four and nine classes on the basis of various parameters for P efficiency as proposed by different researchers. Most genotypes behaved in a similar fashion by different categorization methods and also at different P supply. The method to categorize the genotypes into three classes and plotting them into 9 classes proposed by Gill and his coworkers, is the best to differentiate the minor differences in genotypes. At least three different parameters at both P regimes should be used. The parameters may vary as per objectives of the study and/or growth conditions.

## Introduction

Phosphorus deficiency is a common problem in crop production all over the world. It is estimated that over 40% of world’s cultivated land is short of P for crop production [1]. Phosphorus availability can be problem in both *i*.*e*. alkaline calcareous soils and acidic soils. High pH and higher concentration of CaCO_3_ are responsible for P unavailability in alkaline calcareous soils [2–4] while in acidic soils, higher concentration of Al and Fe declines P availability to plants [5,6]. Moreover, continuously increasing prices, injudicious use of N fertilizers and increased environmental issues of phosphoric fertilizers [7] aggravate the situation manifold and are among the major reasons of low P application in crop production system [1,8]. Rock phosphate ores may get depleted in the near future. The situation impels the invention and adoption of strategies to enhance P acquisition and use by plants for sustainable P management and to breed/produce more P efficient crop plants.

Ortiz-Monasterio and his coworkers [9] suggested phosphorus efficiency as the ability of a genotype to acquire P from soil or growth medium and/or to utilize them in the production of grain and biomass. However, care should be taken while comparing the genetic and physiological components of plants to measure P efficiency. Different methods may yield different P efficiency of the same genotypes under the same environment. The ability of plants to produce yield per unit uptake of P is also termed as P efficiency [10]. This definition would be more promising than total P uptake as sufficient genetic variability exists in biomass production (tall vs dwarf cultivars) [11]. Some researchers focused on root characteristics for P efficiency under low input farming systems [12–14]. However, both root (acquisition) and shoot/grain (internal utilization) should be focused in endeavours aimed at studying P efficiency among cultivars especially under low plant available P environment.

There exists a huge gap between the crop potential and actual production under inapt environments like P deficiency [15]. Wheat is being cultivated on almost every part of the globe, and thus have potential to withstand all types of environmental anomalies. To develop P-efficient wheat cultivars, a basic knowledge of physiological, biochemical and molecular mechanisms is needed. Large genetic variability exists in wheat genome particularly in D genome [16]. The knowledge of such genetic variability may be exploited to produce more P stress tolerance genotypes. However, identification and screening of wheat genotypes responsible for P efficiency is a pre-requisite for such exploitation of genetic variation.

Regarding the efficiency of genotypes against P stress, different scientist proposed different criteria *i*.*e*. total P uptake [14], ratio of dry matter produced at adequate and deficit condition per unit P applied [11] and ratio of physiologically active higher P (Pi) to total P uptake [17,18]. Phosphorus uptake and use efficiency are two distinct characteristics of plants, the first represents the plant’s capacity to take P from soil and the later explains how efficiently the plants utilize the absorbed P to produce biomass.

Our current understanding of P efficiency among wheat genotypes varies with the parameters and methods of P efficiency calculation [17–19]. Therefore, a basic knowledge of different parameters and methods of calculation needs particular attention for better understanding of how P efficiency can be incorporated into assessing germplasm. Two controlled environment studies were conducted to investigate the growth and P uptake of thirty wheat cultivars. This comparison aimed to identify different parameters and methods of P efficiency calculation among wheat genotypes.

## Materials and methods

### Experiment I

The thirty (30) wheat genotypes used in this experiment are presented in Table 1. The seeds of all these genotypes were kindly provided by Wheat Research Institute, Faisalabad, Pakistan.

**Table 1:** Group of wheat genotypes used in study with parentage and the year of release.

Sr. No.	VARIETY/LINE	PARENTAGE	YEAR OF RELEASE
**(I) Twentieth Century Varieties**			
1	T-96725		1911
2	C-591	T9/8B	1934
3	DIRK	FORD/DONDEE	1946
4	C-271	C-230/IP165	1957
5	MEXI PAK	PENJAMO “S”/GABO	1965
6	SA-42	C-271*2//LR64/SON64	1971
7	BLUE SILVER	11.54. 388/AN/3/YT 54/NIOB//LR 64	1971
8	LYP-73	BB/NOR67	1973
9	SANDAL	CNO “S”//SON64/KLRE/3/8156	1973
10	PARI-73	CNO “S”//SON64/KLRE/3/8156	1973
11	LU-26S	BLS/KHUSHAL 69	1978
12	PAK-81	KVZ/BUHO “S”//KAL/BB	1981
13	BARANI-83	BB/GLL/3/GTO/7C//BB/CNO	1983
14	KOHINOOR-83	ORE1.158/FDL//MXFN/TBA/3/COC	1983
15	WADANAK-85	GUL “S”/SNIPE “S”//GDO-VZ449	1985
16	CHAKWAL-86	FLN/ACC//ANA75	1986
17	PASBAN-90	INIA66/A.DISTT//INIA66/3/GEN81	1990
18	INQ-91	WL711/CROW “S”	1991
19	PARWAZ-94	(V5648) CNO “S”/LR64//SON64/3/SON/4/PRL “S”	1994
20	D-97	JO“S”/AA“S”//FG“S”	1998
21	IQBAL-2000	BURGUS/SORT-12-13//KAL/BB/3/PAK81	2000
**(II)Twenty-first Century Varieties**			
22	SH-02	INQ-91/FINK “S”	2002
23	GA-02	DWL5023/SNB//SNB	2002
24	BHAKKAR-02	P102-PIMA//F3.71/TRM/3/PVN-92T001	2002
25	SEHER 06	CHIL/2*STAR/4/BOW/CROW//BUC/PVN/3/2*VEE#10	2006
26	LASANI-08	LUAN/KOHISTAN 97	2008
27	MIRAJ-08	SPARROW/INIA//V-7394/WL 711/3/BAB “S”	2008
28	MILLAT-11	CHENAB2000/INQ.91	2011
29	DARABI-11	HXL 753/2*BAU//PASTOR	2011
30	GALAXY-13	PUNJAB96/87094//MH97	2013

All varieties were officially released for cultivation in Punjab, Pakistan except Dirk, which was imported from Australia

### Plant growth and analysis

A solution culture experiment was conducted in the glasshouse of Institute of Soil and Environmental Sciences (ISES), University of Agriculture Faisalabad, Pakistan. Plants were grown under average temperature ranged from 11°C (night) to 27°C (day), with sun rise at 06:35 h and sun set at17:09 h. Light intensity varied between 400 to 1300 μmol photon m^−2^ s^−1^. Washed river sand in plastic cups was used for seed germination. After twelve days of germination, root systems of seedlings were washed thoroughly with deionized water and uniformly sized seedlings were removed carefully and transported to continuously aerated nutrient solution in polyethylene foil-lined metal tub (200 L capacity) with plants in the holes of the lid supported by foam plugs (randomization was done by using alpha lattice design on computer-based software CropStat 7). Full-strength nutrient solution included micro-elements (Mn SO_4_, 2 μmol L^−1^; (NH_4_)_2_MoO_7_, 0.5 μmol L^−1^; H_3_BO_3_, 25 μmol L^−1^; Cu SO_4_, 1 μmol L^−1^; Zn SO_4_, 2 μmol L^−1^ and Fe-EDTA, 0.1 mmol L^−1^) and macro-elements ((NH_4_)_2_ SO_4_, 0.5 mmol L^−1^; KH_2_ PO_4_, 0.2 mmol L^−1^; K_2_ SO_4_, 4 mmol L^−1^; Ca (NO_3_)_2_, 2mmol L^−1^ and Mg SO_4_, 1 mmol L^−1^;). The plants were grown for 15 d after transplanting with adequate Pi supply (200 μm KH_2_PO_4_). The nutrient solution was replaced with one-week interval for continuous supply of nutrients. The pH of the solutions was adjusted to 6.5 ± 0.05 units daily. On 16^th^ day after transplantation (DAT), two groups of plants were made *i*.*e*. receiving nutrient solution either with 20 μm KH_2_PO_4_ or with 200 μm KH_2_PO_4_ added P.

Prior to harvesting, leaf area meter (AM300) was used to measure leaf area. Plants were harvested 35 DAT and rinsed with distilled water and dried at 70°C until constant dry weights (DWs). Then plant samples were ground and a homogenous portion of ground plant samples were digested by a method proposed by [20] (using di-acid; HNO_3_:HClO_4_ mixture) and P concentration was estimated with the help of colorimetric analysis [21].

Phosphorus utilization efficiencies (PUE) and P stress factor (PSF) were calculated by the following formulae

$$PUE\;(mg\;square\;SDM\;per\;\mu g\;P)=\frac{SDM/GY\;(g)}{P\;concentration\;(\frac{mg}{g})}\times 1000$$

$$PSF\;(\%)=\frac{SDM\;(ad.)-SDM\;(def.)}{SDM\;(ad.)}\times 100$$

Where

SDM (shoot dry matter), GY (grain yield), ad. (adequate; 200 μm KH_2_PO_4_), def. (deficit; 20 μm KH_2_PO_4_)

### Experiment II

#### Treatments, design and growth condition

Twelve wheat genotypes (*i*.*e*. six P efficient; Dirk, Bhakkar-02, Sandal-73, Blue Silver, Pak-81 and D-97 and six P-inefficient; Sehar-06, Pari-73, MaxiPak-65, Glaxy-13, Millat-11 and Iqbal-2000) selected from first experiment were sown in 6 kg soil polythene lined plastic pots. Soil was collected from six feet depth at Soil Science experimental station, UAF. Then soil was air-dried, ground to pass through a 5mm sieve and mixed thoroughly. The texture of the soil was sandy loam [22]. The pH was 7.62 and the EC (electrical conductivity) of the saturated soil paste extract was 2.08 dS m^−1^. Plant available P in the soil was 0.19 mg kg^−1^ soil, measured on UV-visible spectrophotometer (Shimadzu UV-1201-Japan) by Olsen methods of sodium bicarbonate extraction [23]. Soil organic matter was 3.1 g kg^−1^ of the soil [24].

Phosphorus fertilizer was applied at two levels *i*.*e*. deficit (0 mg P kg^-1^ soil) and adequate (30 mg P kg^-1^ soil). The twelve wheat genotypes were arranged by a completely randomized design (CRD) with factorial arrangements in three replicates. Nitrogen (N) and potassium (K) were added @ 70 and 50 mg kg^-1^ soil, respectively as a basal dose. Urea and potassium sulphate were used as a source for N and K, respectively. Mono-ammonium phosphate (MAP) was used as a source of P. The soil in each pot was mixed thoroughly after fertilizer application. Six seeds were grown in each pot and thinned to two plants per pot after germination. Moisture contents at field capacity were maintained by deionized water. The average daily temperature was 23±4˚C (day) and 13±4˚C (night). Light intensity varied between 450 to 1350 μmol photon m^−2^ s^−1^. Plants were harvested at maturity and grains were separated manually. Harvested straw and grain samples were oven dried, with dry weights recorded, ground, digested and analysed for P measurement. Phosphorus efficiency calculation was done in a similar fashion as described in the first experiment.

All the data obtained, was statistically analysed using software; STATISTIX 8.1. Tukeys HSD (Honestly Significance Difference) test was applied to check treatment significance [25].

### Categorization methods

#### Method 1

Wheat genotypes were classified into 3 categories as proposed by Osborne and Rengel [26] and Aziz and his collegues [27]. These categories include I) efficient (E), II) medium (M), III) inefficient (I). The genotypes were assigned as efficient if their mean was > μ+SD, medium if their mean was between μ−SD to μ+SD and inefficient if their mean was <μ-SD. The score assigned 3 to efficient (E), 2 to medium (M) and 1 to in-efficient (I) in respective parameters and cumulative score was counted by summing up the individual scores of different parameters of a genotype.

#### Method 2

According to this method, wheat genotypes were classified into four categorize viz i) efficient and responsive (ER), ii) efficient and non-responsive (ENR), iii) inefficient but responsive (IR) and (iv) inefficient and non-responsive (INR) [28,29]. Efficient means genotypes having dry matter higher than the average dry matter and responsive means genotypes having PUE higher than the average PUE and vice versa.

#### Method 3

A plot is constructed between shoot dry matter/grain yield (x-axis) and P uptake (y-axis). Each axis is divided into three parts (i.e. low, medium and high). The genotypes were assigned as low if their mean was > μ+SD, medium if their mean was between μ−SD to μ+SD and high if their mean was <μ-SD. Finally, wheat genotypes were classified into nine categories i.e. low dry matter-low P (LDM-LP), low dry matter-medium P (LDM-MD), low dry matter-high P (LDM-HP), medium dry matter-low P (MDM-LP), medium dry matter-medium P (MDM-MP), medium dry matter-high P (MDM-HP), high dry matter-low P (HDM-LP), high dry matter-medium P (HDM-MP) and high dry matter-high P (HDM-HP) [30].

## Results

### Growth, biomass and yield production

#### Experiment I

**Fig 1:**
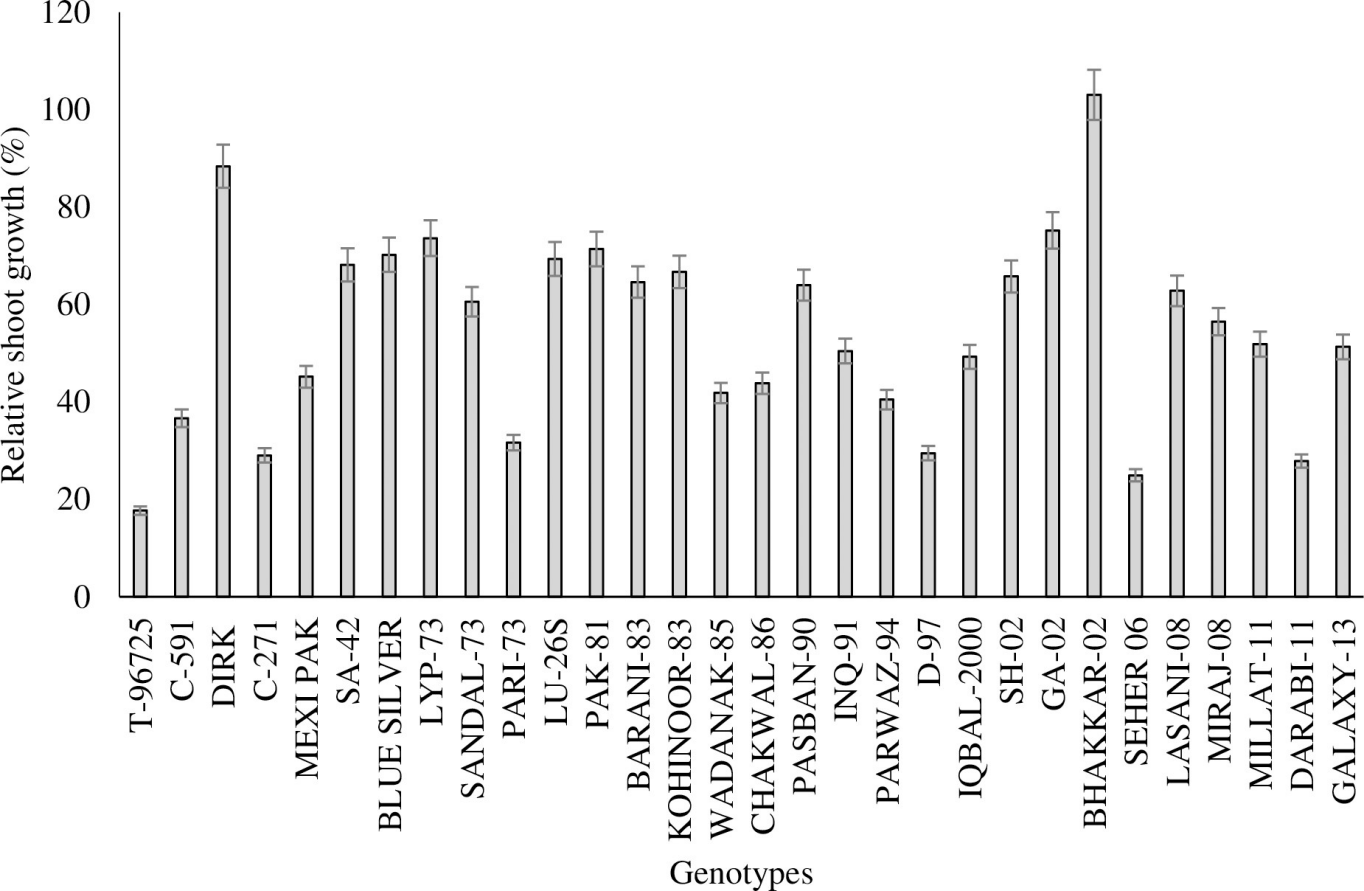
Shoot growth of thirty wheat genotypes grown under P-deficit condition relative to growth at adequate P supply. Bars overlapping with each other do not differ from each other and data are shown as means ± SE of four replicates.

The genotypes varied significantly in growth response at adequate and deficit P levels. A significant (*P<0*.*01*) interactive effect was observed among genotypes and P level with regard to root dry matter (RDM), shoot dry matter (SDM) and root:shoot ratio. In terms of SDM, a general reduction (averaged 50%) was observed with the decrease in P level from 200 μm to 20 μm, however extent of reduction varied among genotypes. The SDM ranged from 0.15 to 2.8 g/plant and from 0.05 to 1.56 g/plant at adequate and deficit P levels, respectively (Table 2). At deficit P level, the maximum SDM was produced by Dirk (1.54 g) and minimum SDM produced by Pari-73 (0.05 g) (S1 Table). The maximum SDM reduction was observed in genotype T-96725 (18%). Growth of genotype Bhakkar-02 was not affected by P deficiency while genotype Dirk produced about 80% of its potential at deficit P supply (Fig 1). The root dry matter (RDM) varied significantly between two P levels among genotypes indicating strong interactive effect of both variables. At deficit P level, the maximum RDM was produced by genotype Dirk (0.81 g/plant) while minimum RDM was produced by genotype Pari-73 (0.02 g/plant). The RDM produced by genotype D-97 at both P levels was statistically at par (0.19 g) (S1 Table). The root depth (measured as distance from root:shoot junction to the root tip of most lengthy root, cm) increased almost 50% by decreasing P level. These results were further supported by increased root:shoot ratio (Table 2). The root:shoot ratio was increased by decreasing P level. At deficit P level the root:shoot ratio was ranged between 0.18 (Millat-11) and 0.53 (T-96725).

**Table 2:** Range of plant height (PH), leaf area, dry matter (DM), root:shoot ratio, P concentration, uptake and utilization efficiency of thirty wheat varieties grown at adequate (200 μm KH_2_PO_4_) and Deficit (20 μm KH_2_PO_4_) P levels under hydroponic condition.

	P levels	
Plant parts/parameters	Adequate	Deficit
Shoot length (cm)	59.6 (20.6–76.5)	48.3 (14.7–72.7)
Root depth (cm)	37.5 (12.8–55.6)	64.4 (12.8–113.1)
Leaf Area (cm^2^)	25.6 (6.20–45.9)	15.8 (5.8–22.11)
**Dry Matter (g plant**^**-1**^**)**		
Shoot	1.01 (0.15–2.80)	0.48 (0.05–1.56)
Root	0.23 (0.05–0.46)	0.17 (0.02–0.81)
Total	1.23 (0.20–3.24)	0.65 (0.06–2.37)
Root:shoot ratio	0.26 (0.15–0.34)	0.35 (0.18–0.53)
P stress factor (%)	─	45.9 (-3.0–82.3)
**P concentration (mg g**^**-1**^ **SDM)**		
Shoot	5.01 (1.08–7.40)	3.92 (0.50–6.93)
Root	2.10 (0.83–6.93)	1.26 (0.63–2.12)
**P uptake (mg P plant**^**-1**^**)**		
Shoot	5.09 (0.31–19.7)	1.94 (0.07–6.51)
Root	0.48 (0.07–1.16)	0.24 (0.01–1.71)
Total	5.57 (0.39–20.6)	2.18 (0.09–8.22)
**P utilization efficiency (mg**^**2**^ **SDM ug**^**-1**^ **P)**		
	265.2 (72.4–1235.8)	218.9 (32.5–803.6)

Values in Parenthesis represents range of the data while the values outside the parenthesis are means of all genotypes

#### Experiment II

The main and interactive effect of genotypes and P levels were significant on yield (Table 3). At P stressed condition, maximum grain yield was observed in genotype Dirk (4.96 g pot^-1^) while the minimum grain yield was observed in genotype Pari-73 (1.46 g pot^-1^) while at adequate P level, maximum and minimum grain yield was observed in genotype Dirk (5.23 g pot^-1^) and genotype Millat-11 (1.71 g pot^-1^), respectively. Maximum and minimum yield reduction was observed in genotype Bhakkar-02 (23%) and Pak-81 (4%), respectively.

**Table 3:** Grain yield and P concentration and P utilization efficiency of twelve selected wheat genotypes at stress and adequate P levels (Adequate; 30 mg P kg^-1^ soil & Deficit; 0 mg P kg^-1^ soil). Values are means ± S.E n = 3.

Parameters	Grain Yield (GY) (g pot^-1^)		Grain P concentration (mg g^-1^)		P utilization efficiency (mg^2^ GY μg^-1^ P)	
Genotypes	Adequate	Deficit	Adequate	Deficit	Adequate	Deficit
DIRK	5.23±0.13	4.96±0.14	3.56±0.07	4.34±0.12	1467.8±32.0	1143.5±36.3
MEXI PAK	3.97±0.13	3.81±0.13	4.63±0.13	3.66±0.15	857.7±9.6	1040.7±17.8
BLUE SILVER	3.52±0.12	2.77±0.12	4.06±0.05	3.54±0.06	781.5±13.3	866.6±15.1
SANDAL-73	3.94±0.14	3.63±0.13	3.83±0.15	3.47±0.11	1028.3±20.3	1047.4±23.9
PARI-73	1.86±0.13	1.31±0.12	3.95±0.14	3.75±0.13	696.2±7.0	331.0±8.2
PAK-81	3.59±0.13	3.45±0.13	4.26±0.16	3.98±0.14	842.9±8.9	694.5±4.8
D-97	3.82±0.15	3.39±0.14	4.09±0.17	3.51±0.14	961.1±29.6	934.9±15.3
IQBAL-2000	2.51±0.14	1.33±0.13	4.41±0.05	4.05±0.08	570.0±9.3	327.8±8.7
BHAKKAR-02	3.65±0.14	2.81±0.14	3.81±0.17	3.45±0.07	814.8±11.4	957.9±8.3
SEHER 06	3.58±0.14	2.85±0.14	4.57±0.14	4.10±0.13	783.4±9.7	695.8±22.9
MILLAT-11	1.71±0.12	1.59±0.13	2.50±0.12	3.00±0.15	635.2±12.3	570.7±2.9
GALAXY-13	3.35±0.13	2.69±0.12	4.01±0.04	3.71±0.06	835.8±19.8	726.4±5.8

HSD_0.05_ Grain yield 0.09; Grain P concentration 0.18; P utilization efficiency 56.2

### Phosphorus concentration and contents

#### Experiment I

The main and interactive effects of P levels and genotypes were significant (p<0.01) on shoot and root P concentration and contents. Under P stress condition, the range of root and shoot P concentration was 0.63 to 2.12 and 0.5 to 6.93 mg P g^-1^ SDM, respectively (Table 2). Maximum shoot P concentration was observed in genotype Barani-83 at both P levels (7.4 and 6.6 mg P g^-1^ SDM at adequate and deficit P levels, respectively) (S2 Table). Maximum reduction in shoot P concentration because of P deficiency, was observed in genotype Seher-06 (from 2.63 to 0.5 mg P g^-1^ SDM). The genotype Seher-06 showed similar trend for root P concentration. Shoot and root P concentration in genotype Blue Silver did not differ with P levels. At deficit P level the maximum and minimum root P concentration was observed in genotype Dirk (2.12 mg P g^-1^ SDM) and genotype Seher-06 (0.63 mg P g^-1^ SDM), respectively. The trend was similar in total P uptake which was ranged from 0.39 to 20.6 mg P plant^-1^ at adequate and from 0.09 to 8.22 mg P plant^-1^ at deficit P levels (Table 2).

#### Experiment II

Grain P concentration ranged from 2.50 mg P g^-1^ grain to 4.63 mg P g^-1^ grain, however, genotypes significantly varied in response. There was mixed response of genotypes in seed P contents under P stress. Only two genotypes (*i*.*e*. Dirk and Millat-11), showed incremental increase in seed P contents under P stressed environment than P sufficient environment (Table 3).

### Phosphorus efficiency

#### Experiment I

The efficiency of any genotype to convert P into dry biomass/grain yield is reflected by P utilization efficiency. The genotypes with less growth reduction under induced P stress are considered more efficient. In both experiments, there were significant differences among genotypes in utilizing P under stress (Tables 2 and 3). The P utilization efficiency (PUE) under P stress ranged from 32.5 to 803.6 mg^2^ SDM μg^-1^ P. The maximum PUE was in the genotype Sandal-73 while the genotype Pari-73 had lowest PUE.

#### Experiment II

The PUE ranged from 300.9 to 1467.8 mg^2^ grain yield μg^-1^ P under P adequate condition and from 369.0 to 1143.5 mg^2^ grain yield μg^-1^ P under P stressed condition (Table 3). Under P deficiency the genotype Dirk showed maximum PUE followed by Sandal-73, Maki Pak and D-97.

### Categorization of genotypes for P efficiency

#### Method 1

The genotypes varied significantly with respect to each parameter under similar circumstances (Table 4). Under deficit P environment, maximum and minimum score was gained by genotype Dirk (14 out of 15) and genotype Seher-06 (7 out of 15), respectively. Most of the high scoring genotypes are efficient in root:shoo ratio. When the response of genotypes under adequate P condition was taken into consideration, quite different results were obtained (Table 5). Genotypes D-97 and Darabi-11 were scored maximum (13 out of 15), while genotype Pari-73 gained minimum point score (6 out of 15).

**Table 4:** Categorization of wheat genotypes (grown at deficit P; 20 μm KH_2_PO_4_) based on their index scores of various parameters into efficient (E), medium (M) and inefficient (I) scoring genotypes.

Parameters	Shoot Dry Matter	Root Dry matter	Root:shoot ratio	P uptake	P utilization efficiency	Total Score out of 15
Genotypes						
T-96725	M	M	E	M	M	11
C-591	M	M	E	M	M	11
DIRK	E	E	E	E	M	14
C-271	M	M	M	E	M	11
MEXI PAK	I	M	M	M	M	9
SA-42	M	M	M	M	M	10
BLUE SILVER	M	M	M	M	E	11
LYP-73	M	M	M	M	M	10
SANDAL-73	M	M	M	M	E	11
PARI-73	I	E	M	I	M	9
LU-26S	M	M	M	M	M	10
PAK-81	M	M	M	E	M	11
BARANI-83	M	M	M	M	M	10
KOHINOOR-83	M	M	M	M	M	10
WADANAK-85	M	M	E	M	M	11
CHAKWAL-86	M	M	M	M	M	10
PASBAN-90	M	M	M	M	M	10
INQ-91	M	M	M	M	M	10
PARWAZ-94	M	M	M	M	M	10
D-97	I	M	M	M	E	10
IQBAL-2000	M	M	M	M	M	10
SH-02	M	M	M	M	M	10
GA-02	M	M	M	M	M	10
BHAKKAR-02	M	M	M	M	E	11
SEHER 06	I	M	I	I	M	7
LASANI-08	M	M	E	M	M	11
MIRAJ-08	M	M	I	E	M	10
MILLAT-11	M	M	I	M	M	9
DARABI-11	M	M	M	M	M	10
GALAXY-13	M	M	M	M	M	10

The genotypes are assigned as efficient if their mean is > μ+SD, medium if their mean is between μ−SD to μ+SD and inefficient if their mean <μ-SD [28, 29]

The scores are giving to each genotype based on its performance in various parameters. The maximum score 15 assigned with 3 to efficient (E), 2 to medium (M) and 1 to in-efficient (I) in respective parameters

**Table 5:** Categorization of wheat genotypes (grown at adequate P; 200 μm KH_2_PO_4_) based on their index scores of various parameters into efficient (E), medium (M) and inefficient (I) scoring genotypes.

Parameters	Shoot Dry Matter	Root Dry matter	Root:shoot ratio	P uptake	P utilization efficiency	Total Score
Genotypes						
T-96725	E	E	I	E	M	12
C-591	E	E	M	M	M	12
DIRK	M	M	M	E	E	12
C-271	E	M	I	E	M	11
MEXI PAK	M	M	M	M	I	9
SA-42	M	M	M	M	I	9
BLUE SILVER	M	M	M	I	E	10
LYP-73	M	M	E	M	I	10
SANDAL-73	M	M	M	I	E	10
PARI-73	I	I	M	I	I	6
LU-26S	M	M	M	M	I	9
PAK-81	M	M	M	M	M	10
BARANI-83	M	M	M	M	I	9
KOHINOOR-83	M	M	M	M	I	9
WADANAK-85	M	M	E	M	I	10
CHAKWAL-86	M	M	M	I	E	10
PASBAN-90	M	M	M	I	M	9
INQ-91	M	M	M	M	M	10
PARWAZ-94	M	M	M	M	I	9
D-97	E	E	M	M	E	13
IQBAL-2000	M	M	M	M	I	9
SH-02	M	M	M	M	I	9
GA-02	M	M	M	M	I	9
BHAKKAR-02	M	I	M	I	E	9
SEHER 06	M	M	M	I	M	9
LASANI-08	M	M	M	M	I	9
MIRAJ-08	M	M	I	E	M	10
MILLAT-11	M	M	M	M	M	10
DARABI-11	E	E	M	E	M	13
GALAXY-13	M	M	E	M	I	10

The genotypes are assigned as efficient if their mean is > μ+SD, medium if their mean is between μ−SD to μ+SD and inefficient if their mean <μ-SD [28, 29]

The scores are giving to each genotype based on its performance in various parameters. The maximum score 15 assigned with 3 to efficient (E), 2 to medium (M) and 1 to in-efficient (I) in respective parameters

The point score at both P levels (i.e. deficit and adequate) were summed up in another table to check the overall performance of genotypes (Table 6). Maximum point score was obtained by genotype Dirk (26 out of 30) followed by genotypes T96725, C-591, D-97 and Darabi-11 (23 each out of 30), while minimum point score was obtained by genotype Pari-73 (15 out of 30) followed by genotype Seher-06 (16 out of 30).

**Table 6:** Scoring of genotypes grown at deficit (P; 20 μm KH_2_PO_4_) and adequate (P; 200 μm KH_2_PO_4_) P supply under hydroponic condition.

Scoring	Score at deficit P level/ out of 15	Score at adequate P level/ out of 15	Total Score/ out of 30
Genotypes			
T-96725	11	12	23
C-591	11	12	23
DIRK	14	12	26
C-271	11	11	22
MEXI PAK	9	9	18
SA-42	10	9	19
BLUE SILVER	11	10	21
LYP-73	10	10	20
SANDAL-73	11	10	21
PARI-73	9	6	15
LU-26S	10	9	19
PAK-81	11	10	21
BARANI-83	10	9	19
KOHINOOR-83	10	9	19
WADANAK-85	11	10	21
CHAKWAL-86	10	10	20
PASBAN-90	10	9	19
INQ-91	10	10	20
PARWAZ-94	10	9	19
D-97	10	13	23
IQBAL-2000	10	9	19
SH-02	10	9	19
GA-02	10	9	19
BHAKKAR-02	11	9	20
SEHER 06	7	9	16
LASANI-08	11	9	20
MIRAJ-08	10	10	20
MILLAT-11	9	10	19
DARABI-11	10	13	23
GALAXY-13	10	10	20

#### Method 2

**Fig 2:**
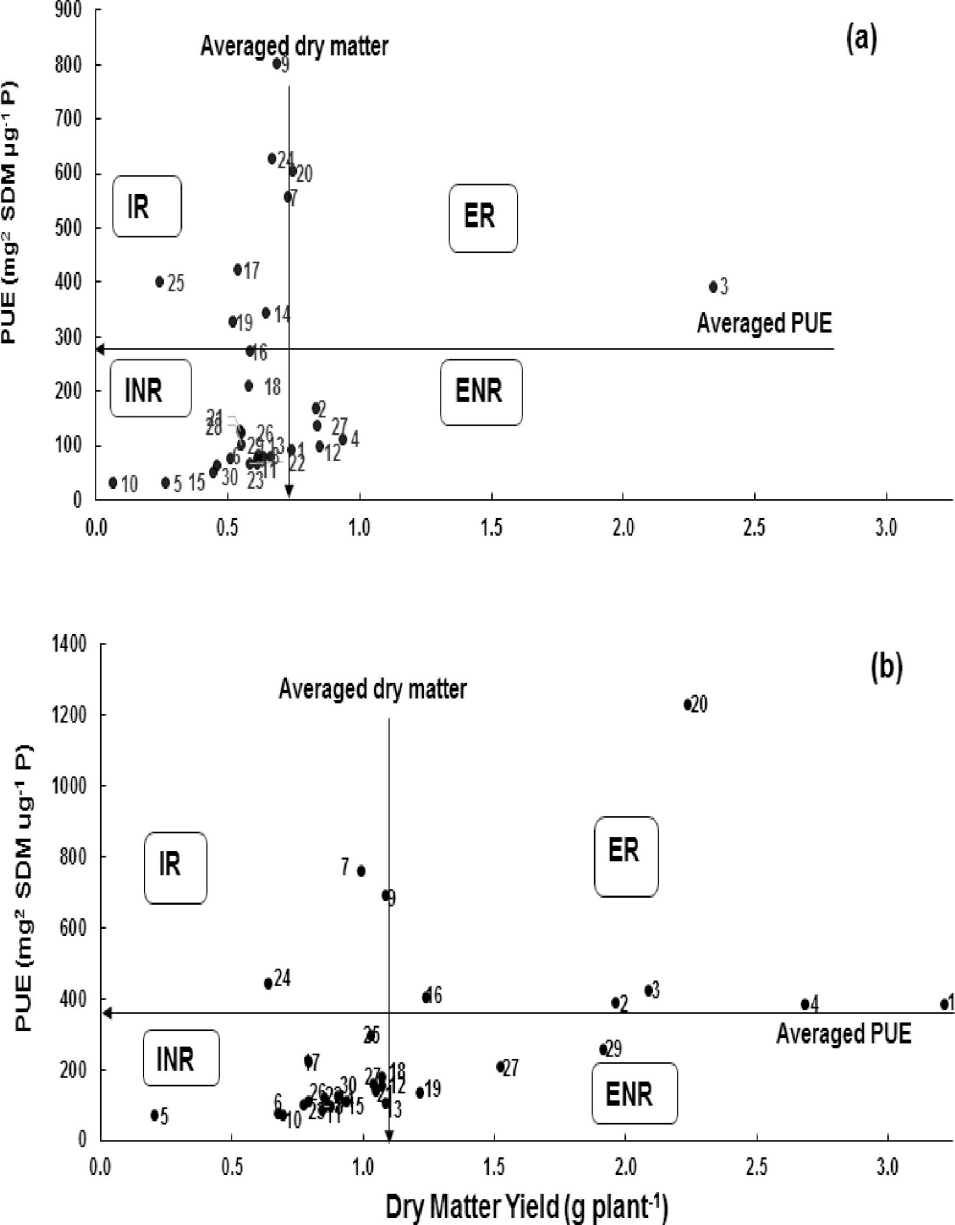
**Classification of wheat genotypes at vegetative growth stage (in hydroponic) for P utilization efficiency a) at deficit P and b) at adequate P supply.** Data are the mean value of four replicates. This categorization divides genotypes into four categories *i*.*e*. efficient and responsive (ER), in-efficient and responsive (IR), efficient and non-responsive (ENR), and in-efficient and non-responsive (INR) [28, 29].

**Fig 3:**
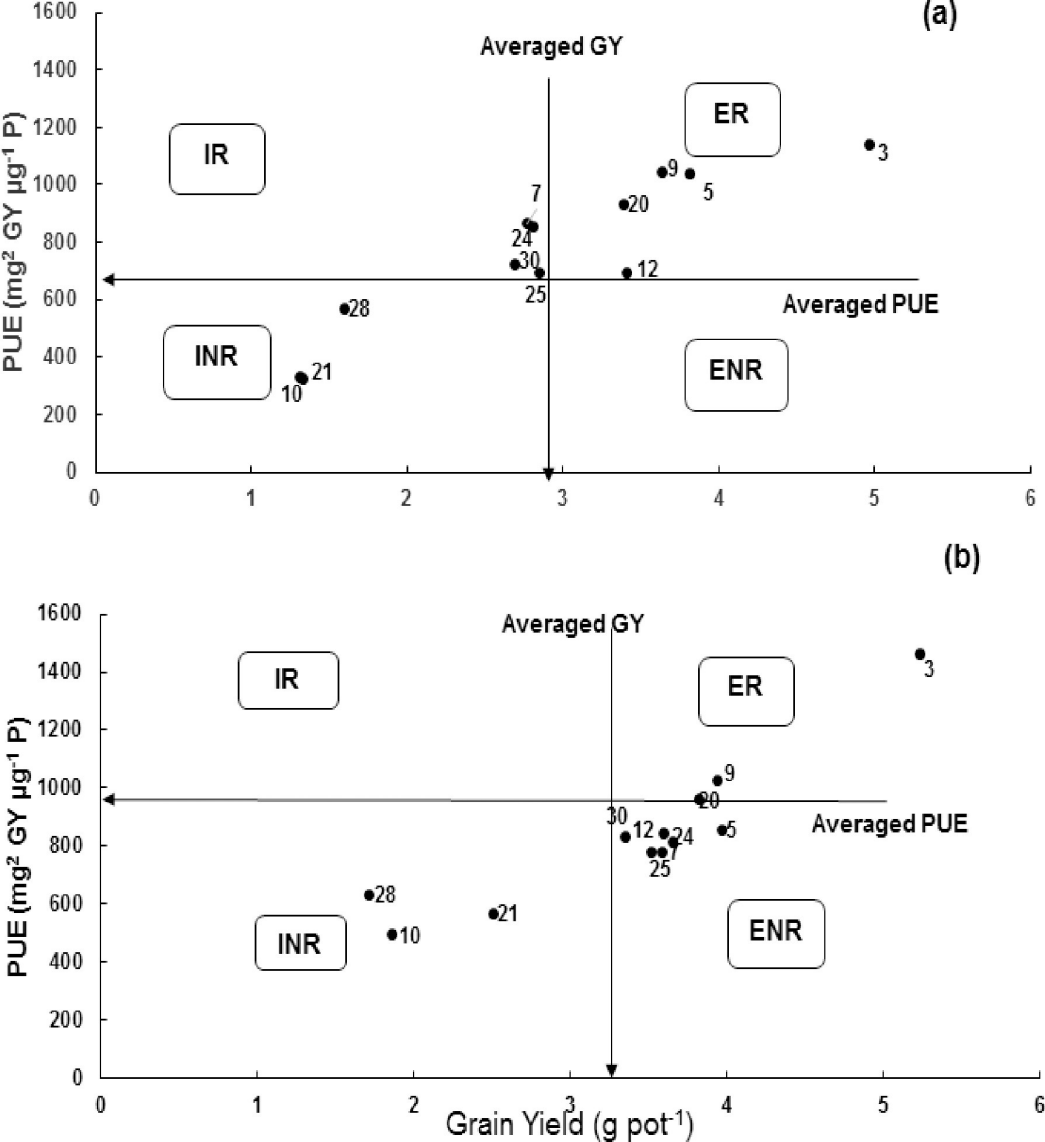
**Classification of wheat genotypes at maturity (in pot culture) for P utilization efficiency a) at deficit P and b) at adequate P supply.** Data are the mean value of four replicates. This categorization divides genotypes into four categories i.e. efficient and responsive (ER), in-efficient and responsive (IR), efficient and non-responsive (ENR), and in-efficient and non-responsive (INR) [28, 29].

Genotypes were categorized at both P levels (i.e. deficit and adequate) (Figs 2 and 3) into four groups. Genotypes T-96725, C-591 and C-271 were efficient and responsive under adequate P supply, while were efficient and non-responsive under deficit P environment (Fig 2). Similarly genotype Chakwal-86 was in-efficient and non-responsive under deficit P, was efficient and responsive under adequate P supply. With regards to grain yield, genotypes Bhakkar-02, Blue Silver, Glaxy-13 and Seher-06 were fall in in-efficient and responsive category under deficit P, while under adequate P, were fall in efficient and non-responsive category (Fig 3).

#### Method 3

**Fig 4:**
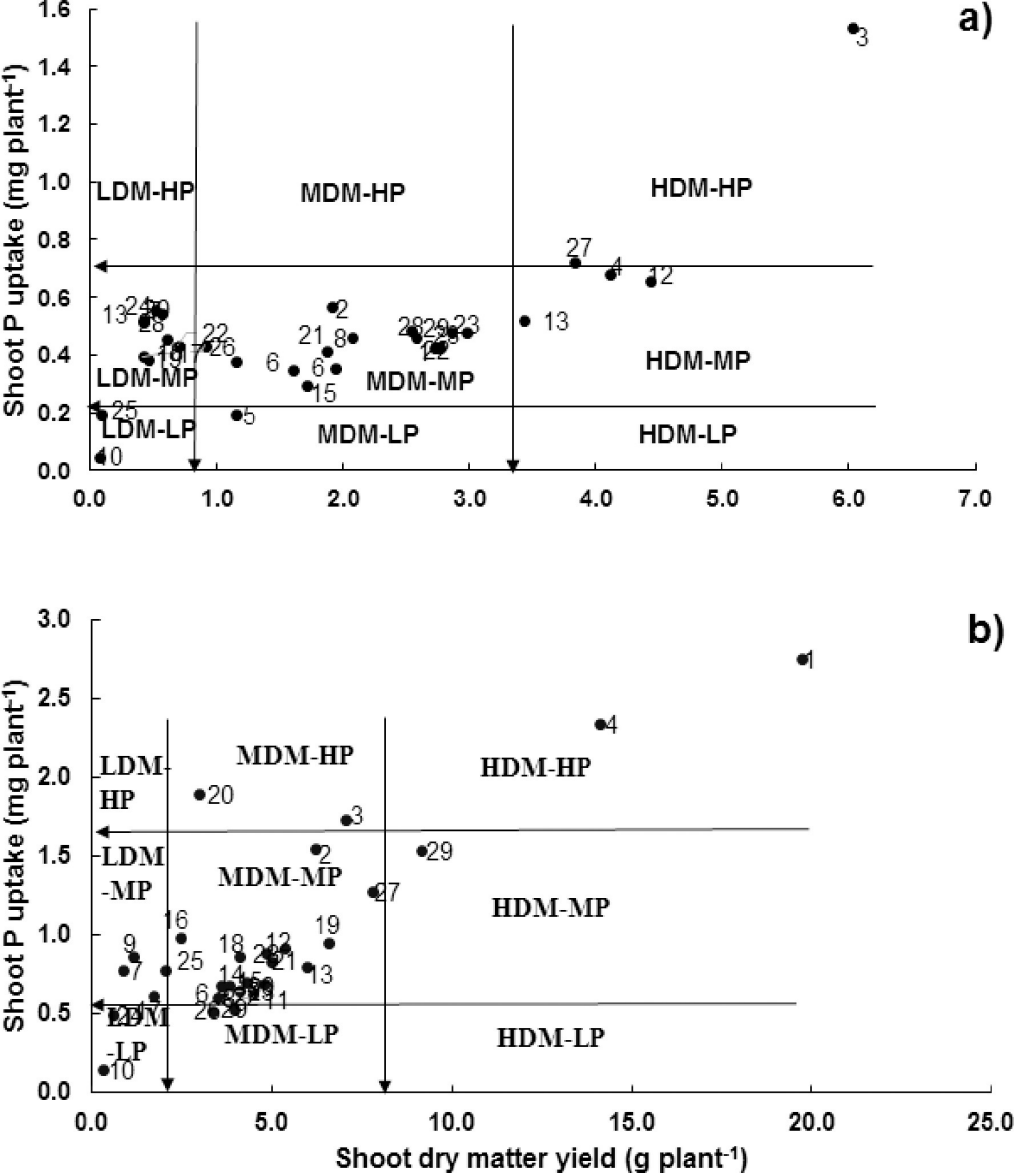
**Classification of wheat genotypes at vegetative growth stage (in hydroponic) for P utilization efficiency a) at deficit P and b) at adequate P supply**. Data are the mean value of four replicates. This categorization divides genotypes into nine categories *i*.*e*. low dry matter-low P (LDM-LP), low dry matter-medium P (LDM-MD), low dry matter-high P (LDM-HP), medium dry matter-low P (MDM-LP), medium dry matter-medium P (MDM-MP), medium dry matter-high P (MDM-HP), high dry matter-low P (HDM-LP), high dry matter-medium P (HDM-MP) and high dry matter-high P (HDM-HP) [30].

This method also yielded significantly different results under both P levels (Fig 4A and 4B). Genotype Miraj-08 was rated as high dry matter-high P (HDM-HP) category under deficit P condition, while it was rated as medium dry matter-medium P (MDM-MP) category under adequate P. Genotype Darabi-11 was high in dry matter production under low P environment while medium in dry matter production under sufficient P environment. Genotypes Dirk and D-97 were medium in shoot P accumulation under deficit P while higher in shoot P accumulation under adequate P condition.

**Fig 5:**
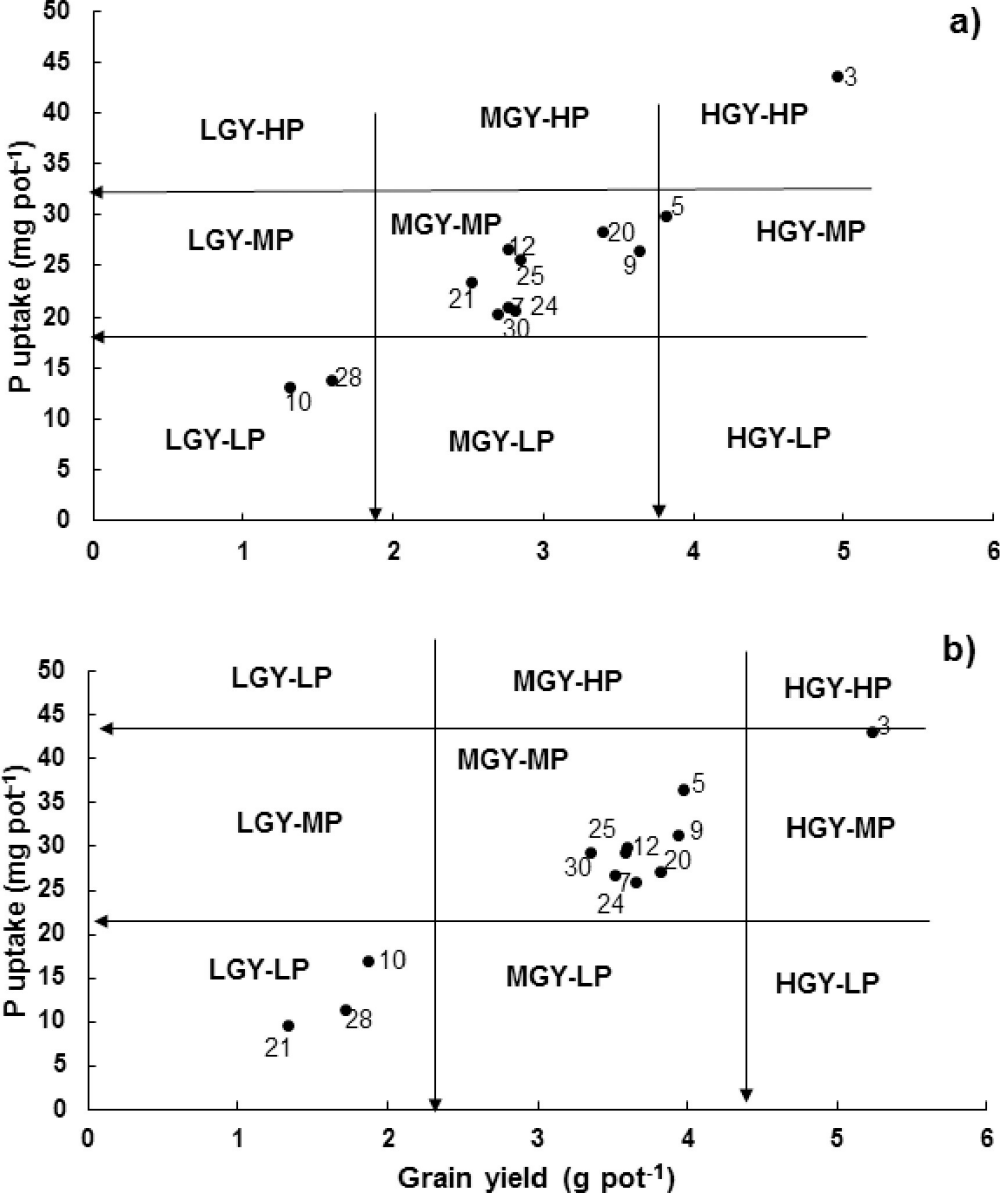
**Classification of wheat genotypes at vegetative growth stage (in pot culture) for P utilization efficiency a) at deficit P and b) at adequate P supply.** Data are the mean value of four replicates. This categorization divides genotypes into nine categories *i*.*e*. low grain yield-low P (LGY-LP), low grain yield-medium P (LGY-MD), low grain yield-high P (LGY-HP), medium grain yield-low P (MGY-LP), medium grain yield-medium P (MGY-MP), medium grain yield-high P (MGY-HP), high grain yield-low P (HGY-LP), high grain yield-medium P (HGY-MP) and high grain yield-high P (HGY-HP) [30].

In terms of grain yield, most of the genotypes categorized similarly under both P levels. However, genotype Iqbal-2000 was categorized as medium grain yield-medium P (MGY-MP) under deficit P condition, while categorized as low grain yield-low P (LGY-LP) under adequate P condition (Fig 5).

## Discussion

### Variation in plant growth, biomass partitioning and yield

The crop species and genotypes among various species differ significantly in growth response under P-deficit and P-adequate condition [27,31–33]. As expected, plant dry biomass production and its partitioning in different organs and PUE of all genotypes varied widely under P-deficit condition (Tables 2 and 3). Such variations are very important for variety development [34] and these attributes can be used in future breeding ventures for producing more P efficient genotypes [35]. The genotypes with more biomass and/or yield (efficient), at both P levels, such as Dirk, D-97, MaxiPak, are desired because they can be fit into the larger range of P environments without compromising the yield [26]. However, dry matter produced or grain yield under P deficient condition may be the most important parameter for screening P efficient genotypes [26,28]. In this study, the genotypes having similar growth and P efficiency at adequate P level, showed large differences in their growth and P efficiency under P deficiency such as Dirk and T-96725, MaxiPak and SA-42, Blue Silver and Pak-81 etc. (S1 and S2 Tables and Table 3).

Biomass partitioning under stress environments is also an important parameter as plants tend to invest more in active plant organs such as young leaves and roots under P stress environment [36–38]. The cultivars rated as efficient also produced maximum root dry matter (S1 Table). Root length and root hair density significantly increases under poor P availability condition, [39] for better P acquisition [40]. Similar trend was observed in most of these genotypes with about 50% increase in root:shoot ratio under P deficiency (Table 2). Investment/allocation of more biomass towards underground part (roots) under P deficiency, in many genotypes (Table 2) indicated that increased root:shoot ratio is an adaptive strategy to invest more on root for acquiring P [27,40].

Correlation between different root traits and P uptake, PUE, shoot dry matter and grain yield was further calculated to see the relationship under P stress (Table 7). A positive and significant correlation (r > 0.5) between root traits and biomass and/or yield related attributes were observed which is thought to be involved in increased production of cytokinins from roots (responsible for biomass partitioning) [41]. This indicates that greater will be the P uptake and its utilization, greater will be the grain yield.

**Table 7:** Correlation matrix showing the relationship of root traits to P uptake (1^st^ experiment) and P uptake and P utilization efficiency to dry matter and grain yield (2^nd^ experiment).

**Experiment I**			
	**Root Depth**	**Root Dry Weight**	**Root:Shoot ratio**
**Root Dry Weight**	0.469419		
**Root:Shoot ratio**	0.355273	0.561736	
**P uptake**	0.397295	0.710814	0.196905
**Experiment II**			
* *	**P uptake**	**P Utilization Efficiency**	**Shoot Dry Weight**
**P Utilization Efficiency**	0.683033		
**Shoot Dry Weight**	0.974186	0.780955	
**Grain Weight/Yield**	0.952696	0.841305	0.973318

### Phosphorus concentration and uptake

Both acquisition and utilization are important, however acquisition is more important for resource poor countries where P application is very low, and lot of added P is fixed causing a serious loss to farmers. On other hand, both P uptake and use are important for farmers applying sufficient or higher doses of P application as in many developed counties [42]. Hence objective should be very clear before categorization of genotypes for P efficiency and onward selection of genotypes for breeding ventures [27].

Phosphorus use efficiency is the dry mass produced per unit P uptake [43]. Sufficient genetic variability occurs among numerous crop species for better P acquisition and utilization under P stressed environment [44] and such differences have already been reported in various genotypes of wheat [18,26,29,45]. Regarding the efficiency of genotypes against P stress, different scientist proposed different criteria *i*.*e*. total P uptake [19], ratio of dry matter produced at adequate and deficit condition per unit P applied [11] and ratio of physiologically active higher P (Pi) to total P uptake [17,18,38]. Phosphorus uptake and use efficiency are two distinct characteristics of plants, the first represents the plant’s capacity to take P from soil and later explains how efficiently the plants has utilized the absorbed P to produce biomass.

### Categorization of genotypes for P efficiency

Low P use, high prices of P fertilizers, fear of depletion of rock P and other geopolitical issues has compelled scientist to produce more P efficient plants and identification of mechanisms aiming at increase P use efficiency in agriculture is first pre-requisite for future breeding ventures. The second prerequisite is to categorize the existing germplasm into various classes based on these parameters/mechanisms. A number of methods and parameters have been proposed for categorization of genotypes with regard to P efficiency [27,29,30,46]. In this study, genotypes were categorized according to different parameters/methods and interestingly genotypes were grouped in different classes when parameter(s) or methods were changed (Tables 4 and 6; Figs 2 and 3).

The genotypes were categorized into three classes [26,27]. Under P deficiency, only one genotype (Dirk) scored 14 out of 15 and rated as efficient. The genotype Seher-06 scored only 7 out of 15, thus rated as inefficient. The genotype Pari-73 was rated as inefficient in 2 parameters/indices and scored 9 (Table 4).

Both Osborne and Rengel [26] and Aziz and his collegues [27] categorized genotypes at only low P conditions, hence this categorization did not answer how well the gentoypes can response when P is available in root medium. As a cultivar may be efficient at low P relative to high P, but may have very low dry matter at adequate P. Hence categorization of genotypes at adequate P is also needed. We categorized genotypes at adequate P level and interestingly some of the genotypes showed quite different response and hence were categorized differently than at deficient P level (Table 5). Under adequate P, the genotype Dharabi-11 scored maximum (13 out of 15) and rated as most efficient while genotype Pari-73 scored minimum (6 out of 15) and rated as inefficient.

Genotypes performing best at both P levels are desired, hence the point scores gained by each genotype were summed and categorization was made. Interestingly most of the genotypes were categorized in the same class, with some exceptions. The genotype Dirk scored maximum (26 out of 30) and genotypes Pari-73 and Seher-2006 scored minimum *i*.*e*. 15 and 16, respectively (Table 6).

Another calculation method was proposed by [28,29] who categorized the wheat genotypes into four groups viz i) efficient and responsive (ER), ii) efficient and non-responsive (ENR), iii) inefficient but responsive (IR) and (iv) inefficient and non-responsive (INR). The most desirable genotypes (*i*.*e*. Dirk and D-97) were grouped as ER because these genotypes produced higher dry biomass under P deficiency while the genotype D-97 was fall under INR category under adequate P supply (Fig 2). Similarly, the genotype Sandal-73 fall in INR category under P stress, in ER category under adequate P supply. Two more graphs were constructed to categorize genotypes with respect to grain yield and P uptake. The genotype Maxi Pak, rated as inefficient and non-responsive at dry biomass production, was rated as efficient and responsive for grain yield under P stress (Fig 3). Hence, classification under adequate P supply is also a need of hour. However, this method has very narrow range between the efficient/responsive and inefficient/non-responsive category *e*.*g*. the genotypes Blue Silver, D-97 and Bhakkar-02 (under P deficiency) and Blue Silver and Sandal 73 (under adequate P supply) were fall near the boundary line of efficient and inefficient (Fig 2). So, this method may not differentiate the genotypes that have narrow range between PUE and dry matter.

The genotypes were classified further into nine categories *i*.*e*. low dry matter-low P (LDM-LP), low dry matter-medium P (LDM-MD), low dry matter-high P (LDM-HP), medium dry matter-low P (MDM-LP), medium dry matter-medium P (MDM-MP), medium dry matter-high P (MDM-HP), high dry matter-low P (HDM-LP), high dry matter-medium P (HDM-MP) and high dry matter-high P (HDM-HP) [30] (Fig 4). Though, this method has more classes to differentiate the minor differences in response of genotypes, yet it has been done only under P deficiency conditions. Hence, the same method was employed at adequate P level.

Under P deficiency, the genotype Dirk was categorized as HDM-HP while under P supply, genotypes T96725 and C-271 were categorized as HDM-HP (Fig 4). Genotype Barani-83 was in higher dry matter-medium P (MDM-MP) category under P deficit condition while, was in medium dry matter-medium P (MDM-MP) category under adequate P condition. This method was further used to categorize genotypes at grain yield response. The genotype Dirk rated as HGY-HP at both P levels (Fig 5). The genotype Iqbal-2000 was rated as MGY-MP under P deficiency while rated as LGY-LP under P supply condition.

## Conclusion

Categorization of existing germplasm for P efficiency is need for agriculture systems and also for breeding ventures to produce more P efficient plants. Three categorization/calculations were employed to group 30 wheat genotypes into various classes as proposed by various scientists. All calculations systems have some (dis)/advantages as discussed above. The method to categorize the genotypes into three classes viz in-efficient, medium and efficient and plotting them into 9 classes proposed by Gill and his coworkers is good enough to differentiate the minor differences in genotypes. However, this only takes into account two parameters viz dry matter (/grain yield) and P uptake. Similar method considering at least four parameters gave much better categorization (Tables 4 and 5). Moreover, the categorization should be done on both low P and high P conditions (Table 6).

## Supporting information

S1 Table Plant dry matter yield, root:Shoot ratio and phosphorus stress factor of thirty wheat genotypes at adequate and inadequate P levels. (DOCX)

S2 Table Plant (shoot and root) P concentration and P utilization efficiency of thirty wheat genotypes at adequate and inadequate P levels. (DOCX)
