# Supplementary material for: Categorization of wheat genotypes for phosphorus efficiency
Source: PLoS One. 2018 Oct 17;13(10):e0205471. doi: 10.1371/journal.pone.0205471 (PMC6192622; doi:10.1371/journal.pone.0205471)
Supplement: S1 Table — (DOCX) [file pone.0205471.s001.docx]

**S1 Table. Plant dry matter yield, root:shoot ratio and phosphorus stress factor of thirty wheat genotypes at adequate and inadequate P levels.**

| **Parameters** | **Shoot Dry Matter**  **(g)** | | **Root Dry Matter**  **(g)** | | **Root:Shoot ratio** | | **Plant Stress Factor**  **(%)** |
| --- | --- | --- | --- | --- | --- | --- | --- |
| **Genotypes** | **Adequate** | **Deficit** | **Adequate** | **Deficit** | **Adequate** | **Deficit** |  |
| T-96725 | 1.75±0.19 | 0.49±0.07 | 0.46±0.03 | 0.26±0.03 | 0.17±0.01 | 0.53±0.08 | 82.30 |
| C-591 | 1.56±0.07 | 0.57±0.06 | 0.41±0.03 | 0.26±0.02 | 0.26±0.03 | 0.46±0.05 | 63.34 |
| DIRK | 1.74±0.04 | 1.54±0.04 | 0.35±0.01 | 0.81±0.02 | 0.20±0.01 | 0.52±0.05 | 11.65 |
| C-271 | 2.34±0.06 | 0.68±0.05 | 0.34±0.03 | 0.26±0.04 | 0.15±0.01 | 0.38±0.05 | 70.97 |
| MEXI PAK | 0.54±0.05 | 0.19±0.04 | 0.16±0.01 | 0.07±0.02 | 0.29±0.02 | 0.36±0.09 | 63.55 |
| SA-42 | 0.52±0.03 | 0.35±0.03 | 0.16±0.03 | 0.11±0.03 | 0.30±0.04 | 0.30±0.06 | 31.88 |
| BLUE SILVER | 0.78±0.04 | 0.55±0.03 | 0.21±0.04 | 0.18±0.03 | 0.27±0.04 | 0.33±0.07 | 29.81 |
| LYP-73 | 0.63±0.06 | 0.46±0.06 | 0.22±0.02 | 0.16±0.02 | 0.34±0.02 | 0.34±0.06 | 26.40 |
| SANDAL-73 | 0.86±0.05 | 0.52±0.05 | 0.22±0.03 | 0.16±0.01 | 0.26±0.04 | 0.31±0.05 | 39.42 |
| PARI-73 | 0.15±0.05 | 0.05±0.02 | 0.05±0.02 | 0.02±0.01 | 0.33±0.03 | 0.32±0.02 | 68.33 |
| LU-26S | 0.69±0.05 | 0.48±0.04 | 0.18±0.02 | 0.16±0.01 | 0.26±0.01 | 0.32±0.04 | 30.69 |
| PAK-81 | 0.92±0.05 | 0.66±0.06 | 0.15±0.01 | 0.19±0.05 | 0.16±0.01 | 0.29±0.06 | 28.65 |
| BARANI-83 | 0.80±0.08 | 0.52±0.03 | 0.28±0.04 | 0.14±0.03 | 0.34±0.02 | 0.27±0.04 | 35.40 |
| KOHINOOR-83 | 0.68±0.05 | 0.46±0.03 | 0.17±0.01 | 0.19±0.02 | 0.25±0.01 | 0.41±0.04 | 33.33 |
| WADANAK-85 | 0.70±0.05 | 0.29±0.03 | 0.23±0.02 | 0.15±0.03 | 0.33±0.01 | 0.50±0.06 | 58.16 |
| CHAKWAL-86 | 0.99±0.06 | 0.43±0.06 | 0.25±0.02 | 0.15±0.03 | 0.25±0.03 | 0.35±0.04 | 56.20 |
| PASBAN-90 | 0.62±0.04 | 0.40±0.07 | 0.17±0.05 | 0.14±0.04 | 0.27±0.07 | 0.36±0.04 | 36.03 |
| INQ-91 | 0.86±0.04 | 0.44±0.06 | 0.21±0.02 | 0.15±0.02 | 0.24±0.03 | 0.33±0.03 | 49.57 |
| PARWAZ-94 | 0.96±0.05 | 0.39±0.02 | 0.26±0.06 | 0.13±0.01 | 0.27±0.06 | 0.34±0.05 | 59.53 |
| D-97 | 1.89±0.40 | 0.56±0.10 | 0.34±0.03 | 0.19±0.02 | 0.19±0.05 | 0.35±0.09 | 70.54 |
| IQBAL-2000 | 0.84±0.07 | 0.41±0.04 | 0.21±0.04 | 0.13±0.03 | 0.25±0.05 | 0.32±0.06 | 50.75 |
| SH-02 | 0.65±0.06 | 0.43±0.05 | 0.22±0.03 | 0.16±0.02 | 0.33±0.03 | 0.37±0.03 | 34.23 |
| GA-02 | 0.64±0.04 | 0.48±0.05 | 0.15±0.01 | 0.13±0.02 | 0.24±0.04 | 0.28±0.06 | 24.80 |
| BHAKKAR-02 | 0.50±0.04 | 0.51±0.04 | 0.14±0.01 | 0.15±0.02 | 0.28±0.02 | 0.30±0.02 | -3.02 |
| SEHER 06 | 0.78±0.29 | 0.20±0.03 | 0.24±0.09 | 0.05±0.01 | 0.31±0.02 | 0.23±0.03 | 75.08 |
| LASANI-08 | 0.61±0.03 | 0.38±0.06 | 0.17±0.02 | 0.17±0.03 | 0.28±0.04 | 0.46±0.08 | 37.19 |
| MIRAJ-08 | 1.28±0.04 | 0.72±0.19 | 0.25±0.04 | 0.12±0.03 | 0.19±0.03 | 0.18±0.07 | 43.53 |
| MILLAT-11 | 0.89±0.46 | 0.46±0.06 | 0.15±0.04 | 0.09±0.03 | 0.18±0.05 | 0.20±0.05 | 48.17 |
| DARABI-11 | 1.54±0.06 | 0.43±0.03 | 0.38±0.02 | 0.18±0.01 | 0.25±0.01 | 0.43±0.04 | 72.15 |
| GALAXY-13 | 0.68±0.06 | 0.35±0.04 | 0.22±0.02 | 0.16±0.04 | 0.32±0.02 | 0.44±0.08 | 48.72 |

HSD_0.05_ Shoot Dry Matter 0.30; Root Dry Matter 0.09; Root: Shoot 0.14

Values are means ± S.E n=4; Adequate; 200 µm KH_2_PO_4_, Deficit; 20 µm KH_2_PO_4_
