# Supplementary material for: Categorization of wheat genotypes for phosphorus efficiency
Source: PLoS One. 2018 Oct 17;13(10):e0205471. doi: 10.1371/journal.pone.0205471 (PMC6192622; doi:10.1371/journal.pone.0205471)
Supplement: S2 Table — (DOCX) [file pone.0205471.s002.docx]

**S2 Table.** **Plant (shoot and root) P concentration and P utilization efficiency of thirty wheat genotypes at adequate and inadequate P levels.**

| **Parameters** | **Shoot P concentration**  **(mg g^-1^)** | | **Root P concentration**  **(mg g^-1^)** | | **P utilization efficiency**  **(mg^2^ SDM µg^-1^ P)** | |
| --- | --- | --- | --- | --- | --- | --- |
| **Genotypes** | **Adequate** | **Deficit** | **Adequate** | **Deficit** | **Adequate** | **Deficit** |
| T-96725 | 7.15±0.40 | 5.20±0.26 | 2.05±0.26 | 1.05±0.24 | 386.1±33.6 | 93.5±9.1 |
| C-591 | 3.98±0.22 | 3.38±0.17 | 1.78±0.34 | 1.35±0.29 | 391.6±15.2 | 169.6±23.0 |
| DIRK | 4.05±0.25 | 3.92±0.31 | 2.63±0.17 | 2.12±0.13 | 429.1±2.10 | 391.4±29.1 |
| C-271 | 6.03±0.35 | 6.05±0.39 | 1.83±0.25 | 1.34±0.39 | 389.9±28.1 | 113.0±13.4 |
| MEXI PAK | 7.40±0.50 | 5.95±0.64 | 1.98±0.28 | 1.28±0.10 | 72.4±5.5 | 33.1±7.7 |
| SA-42 | 6.48±0.55 | 5.53±0.31 | 2.15±0.21 | 1.23±0.29 | 80.5±10.1 | 64.1±8.7 |
| BLUE SILVER | 1.08±0.31 | 1.05±0.31 | 0.83±0.19 | 0.88±0.31 | 764.5±190.7 | 557.7±167.0 |
| LYP-73 | 7.18±0.25 | 5.58±0.39 | 2.45±0.17 | 1.58±0.13 | 87.1±8.7 | 82.3±6.5 |
| SANDAL-73 | 1.33±0.35 | 0.85±0.53 | 1.25±0.13 | 1.38±0.36 | 694.5±130.6 | 803.6±131.0 |
| PARI-73 | 2.10±0.16 | 1.58±0.40 | 1.48±0.17 | 0.89±0.17 | 72.5±22.8 | 32.5±12.8 |
| LU-26S | 6.90±0.28 | 5.98±0.38 | 1.53±0.43 | 1.00±0.22 | 100.7±10.6 | 80.7±9.3 |
| PAK-81 | 5.80±0.34 | 6.70±0.37 | 1.95±0.26 | 1.58±0.15 | 160.1±15.9 | 98.6±9.1 |
| BARANI-83 | 7.40±0.23 | 6.60±0.29 | 2.23±0.28 | 1.18±0.40 | 108.9±11.6 | 78.9±6.3 |
| KOHINOOR-83 | 5.60±0.44 | 1.35±0.21 | 6.93±0.53 | 1.05±0.49 | 122.8±16.6 | 343.8±60.4 |
| WADANAK-85 | 6.08±0.28 | 5.80±0.26 | 2.08±0.42 | 1.43±0.25 | 116.2±7.5 | 50.9±4.8 |
| CHAKWAL-86 | 2.48±0.40 | 1.60±0.28 | 3.30±0.37 | 1.43±0.22 | 409.6±90.6 | 275.0±48.8 |
| PASBAN-90 | 2.78±0.37 | 1.10±0.50 | 3.28±0.43 | 1.28±0.17 | 224.1±16.6 | 424.6±92.5 |
| INQ-91 | 4.78±0.35 | 2.10±0.26 | 2.35±0.19 | 1.43±0.19 | 181.6±19.9 | 209.4±37.6 |
| PARWAZ-94 | 6.84±0.56 | 1.19±0.18 | 3.15±0.21 | 2.00±0.26 | 140.5±9.9 | 329.2±42.3 |
| D-97 | 1.55±0.24 | 0.93±0.13 | 2.03±0.38 | 1.45±0.19 | 1235.8±297.7 | 606.4±113.1 |
| IQBAL-2000 | 5.98±0.61 | 6.93±0.92 | 1.30±0.34 | 1.33±0.29 | 141.4±19.1 | 67.0±23.5 |
| SH-02 | 6.28±0.36 | 6.50±0.29 | 1.23±0.28 | 1.12±0.19 | 104.1±13.7 | 66.1±9.4 |
| GA-02 | 5.70±0.29 | 6.23±0.38 | 1.33±0.21 | 0.98±0.15 | 111.6±8.7 | 76.8±7.4 |
| BHAKKAR-02 | 1.15±0.21 | 0.83±0.10 | 1.30±0.37 | 1.20±0.26 | 447.8±114.7 | 627.0±79.6 |
| SEHER 06 | 2.63±0.15 | 0.50±0.08 | 1.45±0.13 | 0.63±0.22 | 299.4±89.9 | 400.4±94.9 |
| LASANI-08 | 5.78±0.30 | 3.08±0.28 | 3.40±0.37 | 1.28±0.15 | 105.1±10.7 | 125.5±30.4 |
| MIRAJ-08 | 6.10±0.65 | 5.35±0.33 | 1.95±0.21 | 1.40±0.36 | 210.7±21.5 | 135.8±41.1 |
| MILLAT-11 | 5.45±0.29 | 4.48±0.31 | 1.43±0.15 | 1.11±0.28 | 163.2±51.1 | 102.5±8.1 |
| DARABI-11 | 5.98±0.74 | 6.38±0.33 | 1.35±0.24 | 1.05±0.20 | 260.4±39.7 | 67.3±6.9 |
| GALAXY-13 | 5.23±0.25 | 4.60±0.14 | 1.08±0.28 | 0.77±0.10 | 130.7±11.5 | 76.3±11.1 |

HSD_0.05_ Shoot P concentration 0.41; Root P concentration 0.20; P utilization efficiency 66.5

Values are means ± S.E n=4, Adequate; 200 µm KH_2_PO_4_ & Deficit; 20 µm KH_2_PO_4_
